# Chondromyxoid Fibroma of Distal Phalanx of the Great Toe: A Rare Clinical Entity

**DOI:** 10.7759/cureus.7133

**Published:** 2020-02-28

**Authors:** Nagashree Vasudeva, Chinta Shyam Kumar, Ch R Ayyappa Naidu

**Affiliations:** 1 Orthopaedics, Siddhartha Medical College, Vijayawada, IND

**Keywords:** chondromyxoid fibroma, benign bone tumor, lytic lesion, bone grafting

## Abstract

Chondromyxoid fibroma is a rare benign tumor of cartilaginous origin with myxoid and fibrous components. It accounts for approximately 1% of bone tumors. Metaphysis of long bones is the most common location of this tumor. However, there a few case reports of this tumor arising from epiphysis of short tubular bones of the hand and feet.

An 11-year-old girl presented to our OPD with complaints of pain and a gradually progressive swelling of the right great toe. On examination, the swelling was diffuse with no signs of inflammation. X-ray examination revealed a well-defined, longitudinally oval lytic lesion in the right distal phalanx of great toe, involving the growth plate and, eroding the medial cortex. Computed tomography (CT) scan did not show any evidence of calcification, septations or involvement of soft tissue. Open biopsy and curettage was done and the specimen was sent for histopathological examination. Histopathological examination (HPE) showed a lobular pattern consisting of myxomatous stroma and immature cartilaginous cells in lacunae. The lobules were separated by fibrous septae. It was reported to be Chondromyxoid fibroma. The patient presented six months later with persisting pain and X-ray showed recurrence of the tumor. Hence, complete excision of the tumor was done and the defect was filled using synthetic bone graft. At six months follow up, the patient did not complain of pain and X-rays showed signs of bone formation with incorporation of the graft.

Chondromyxoid fibroma is a low grade tumor, which may demonstrate nuclear atypia histologically and mimic chondrosarcoma. Differentiating these two is of paramount importance to avoid over-diagnosis and aggressive treatment. Recurrence is common with marginal excision and especially in younger patients like in our case. Complete resection is the mainstay of management. Long-term follow up of patients is necessary to watch for malignant transformation, a rare complication. Chondromyxoid fibroma is an extremely rare neoplasm of bone. There are no specific radiologic features, and histopathology provides a definitive diagnosis. It should be considered in differential diagnosis of lytic lesion, and differentiated from other tumors, especially from chondrosarcoma to treat the patient appropriately.

## Introduction

Chondromyxoid fibroma is the rarest benign tumor of cartilaginous origin characterized by incomplete cartilage differentiation. It accounts for less than 1% of all bone tumors [1]. It is characterized by a lobulated pattern of spindle shaped fibrous cells in immature chondroid and myxoid stroma [2]. It is frequently found in the metaphysis of long bones with proximal tibia being the commonest site. Short tubular bones of hand and feet are infrequent sites, and toes account for less than 5% of the tumors [3]. Less than 20 cases of chondromyxoid fibroma in the great toe have been reported. We present a case of chondromyxoid fibroma in an 11-year-old girl involving the distal phalanx of the great toe and its management for its rare anatomic site of origin and age of presentation.

## Case presentation

**Figure 1:**
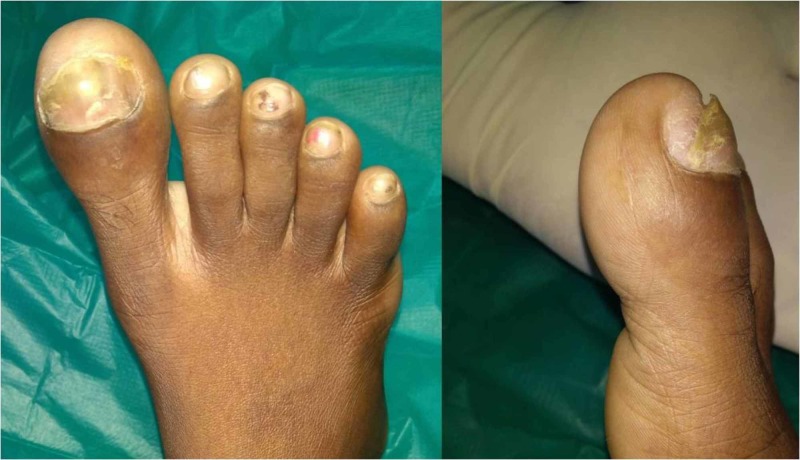
Clinical picture of the right great toe swelling

An 11-year-old girl presented to our OPD with complaints of pain and a gradually progressive swelling of the right great toe for six months. The pain was dull aching of mild intensity, without any diurnal variation, and associated with difficulty while walking. On examination, there was diffuse swelling of the great toe as shown in Figure 1. There was no local rise of temperature, however tenderness was present. The skin over the toe was normal. Range of movements of the distal interphalangeal joint were painful.

**Figure 2:**
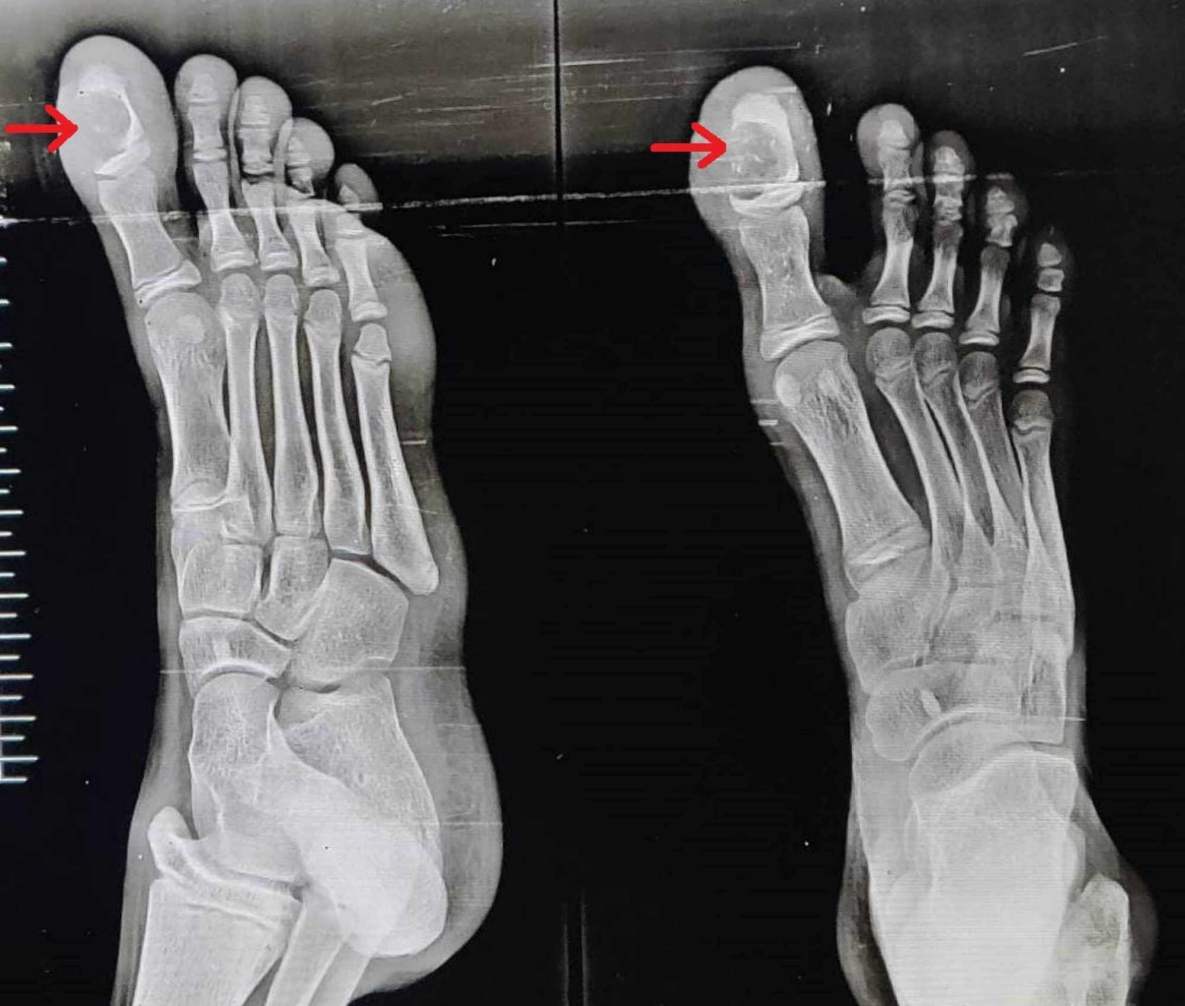
Anteroposterior and lateral view X-rays of the right foot The image shows the lytic lesion in the great toe

**Figure 3:**
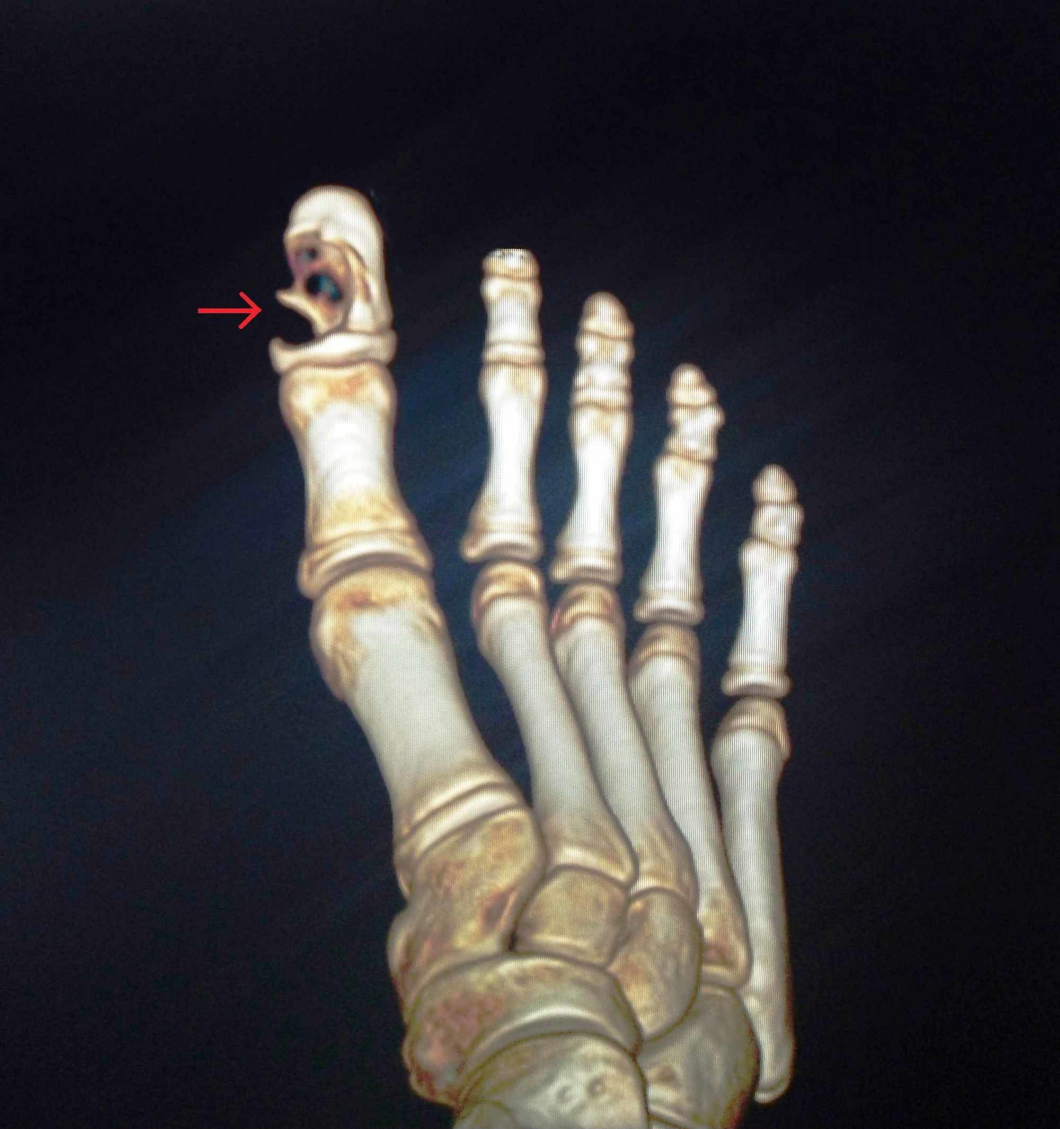
3D-reconstructed computed tomography image of the right foot The image shows lytic lesions with internal septations

X-ray examination revealed a longitudinally oval lytic lesion in the right distal phalanx of great toe, involving almost the entire shaft, abutting the growth plate and eroding the medial cortex, “hemispheric bite like lesion as shown in Figure 2.” The margins were well defined, and there was no articular involvement. Computed tomography (CT) scan showed a well defined lytic lesion in the distal phalanx of great toe with internal septations, without any evidence of calcification or involvement of soft tissue (Figure 3). There was no periosteal reaction.

**Figure 4:**
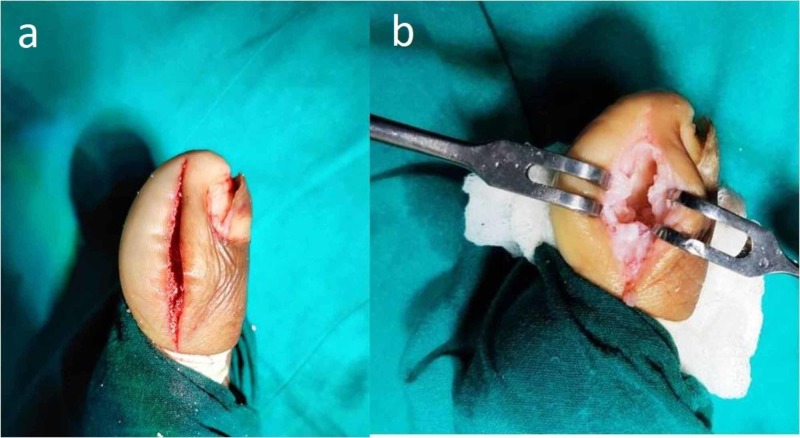
Approach and intra-operative findings (a) shows skin incision of the medial approach; (b) shows cavity of the lytic lesion

With these findings, the patient underwent open biopsy and curettage of the lesion. Medial approach to the distal phalanx was used. Intraoperatively, there was no involvement of the soft tissue. The lesion and the walls of the cavity were excised until healthy cortical bone was seen (Figure 4).

**Figure 5:**
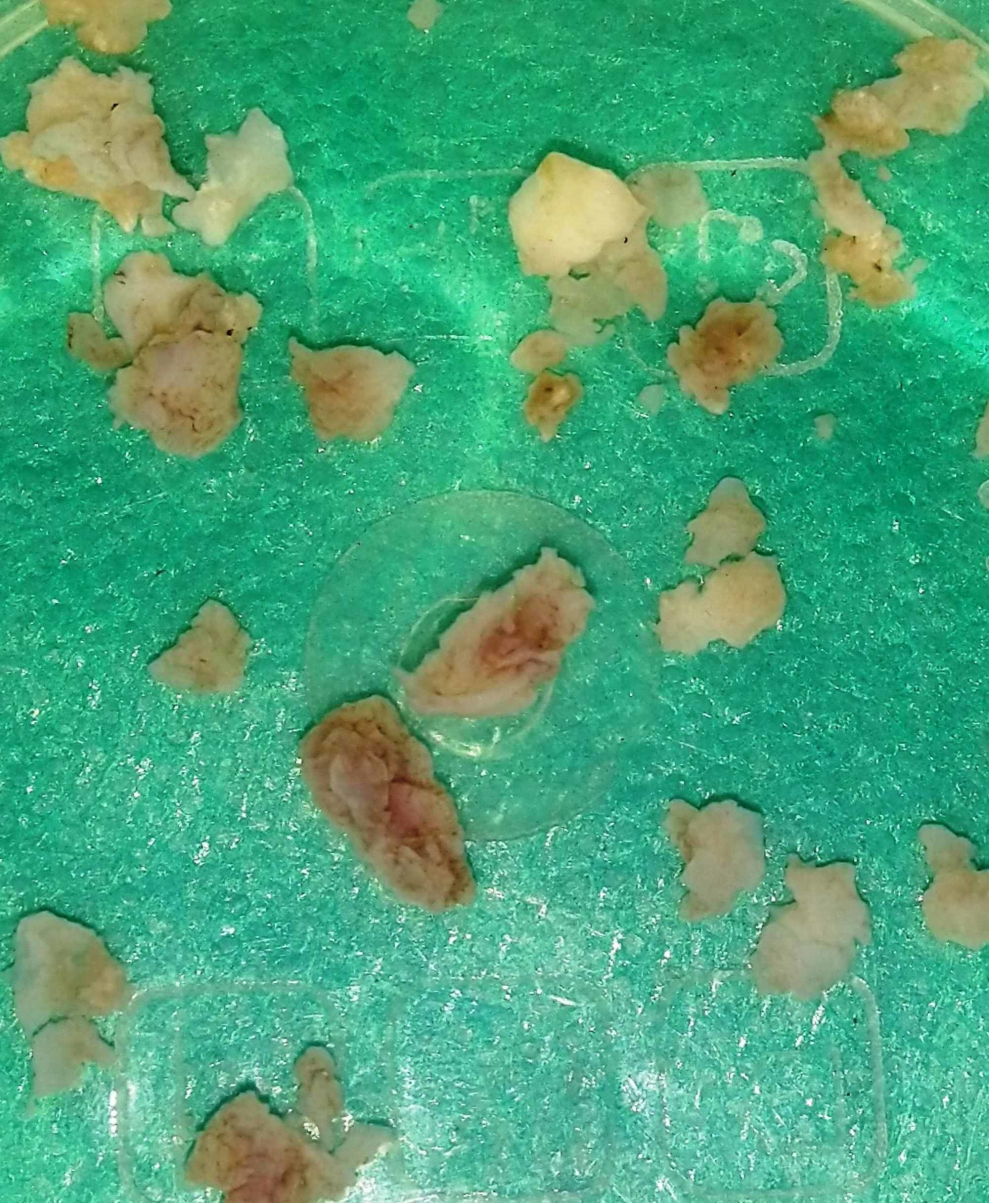
Gross specimen of the lesion

Grossly, the curetted specimen consisted of multiple irregular tissue fragments, which were grey-white and pale brown in color as shown in Figure 5. It was firm in consistency. The specimen was sent for histopathological examination (HPE).

**Figure 6:**
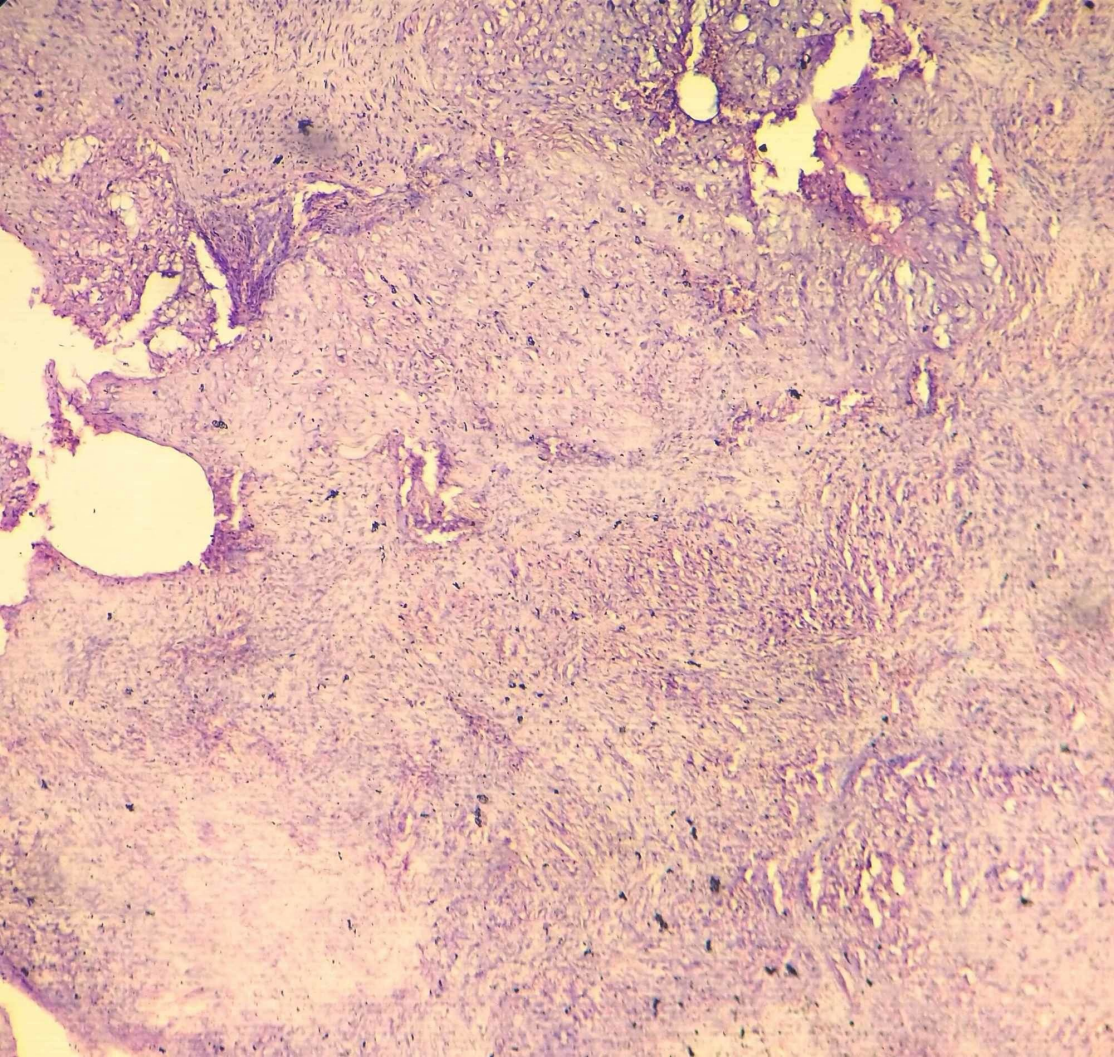
Histopathology of the lesion The image shows hypocellular lobules separated by hypercellular fibrous septa

**Figure 7:**
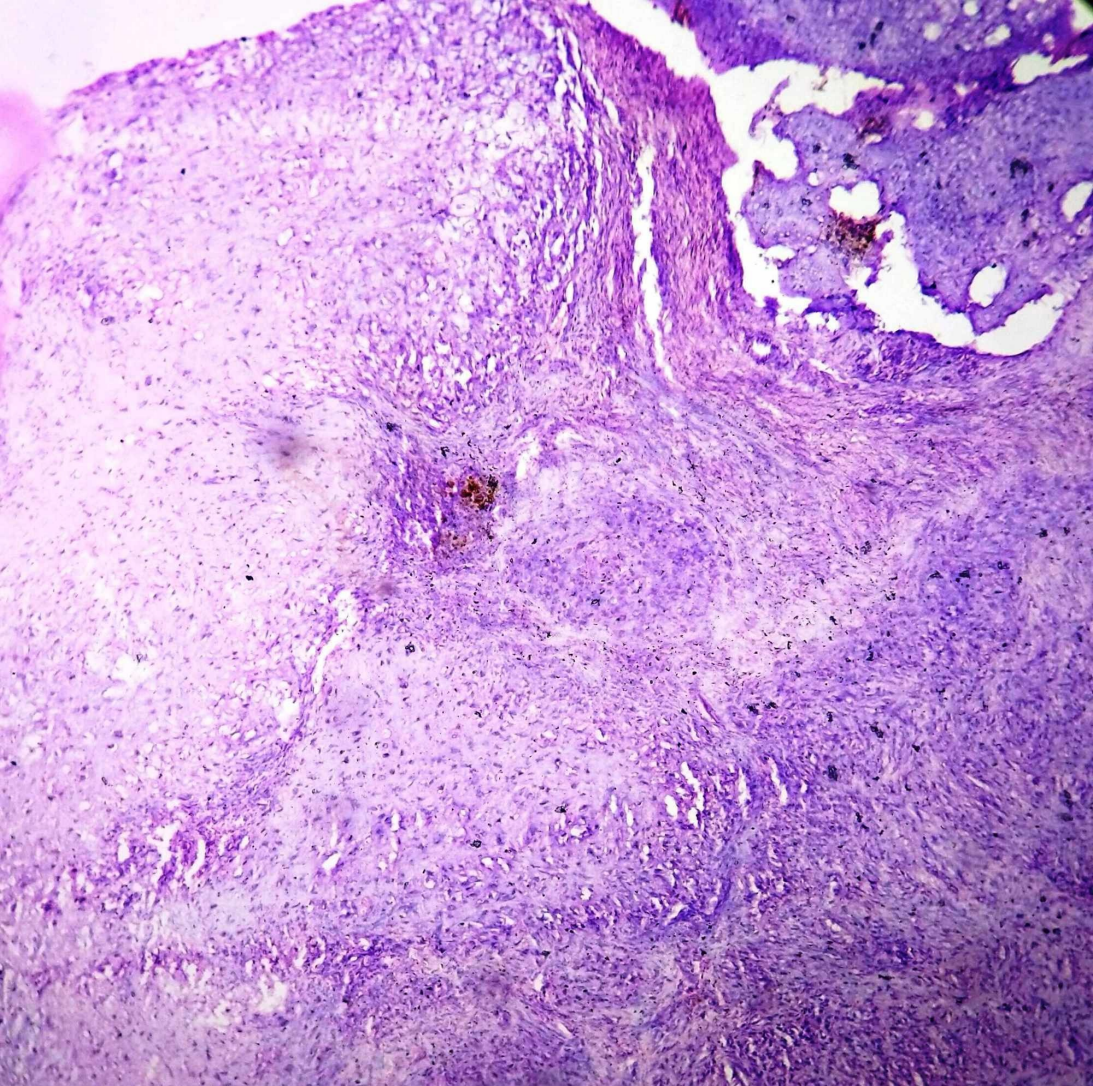
Histopathology of the lesion The image shows chondroid and myxoid stroma with spindle shaped cells in the fibrous septa

**Figure 8:**
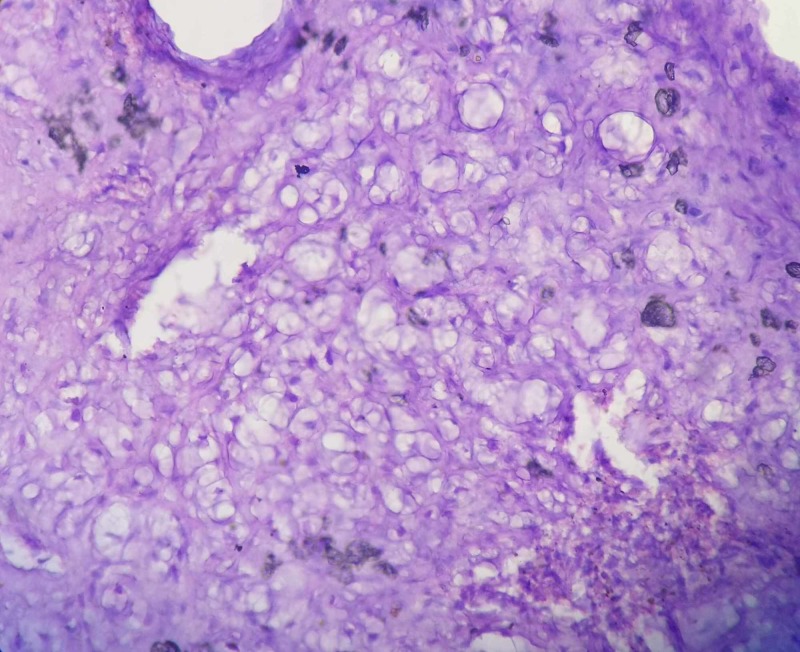
High power field of the histopathology The section shows immature cartilage cells in the lacunae

HPE showed a lobular pattern consisting of myxomatous stroma and immature cartilaginous cells in lacunae. Spindle shaped cells with hyperchromatic nuclei were dispersed in the myxoid matrix. The lobules were separated by hypercellular fibrous septae (Figures 6-8). Giant cells and calcification were not seen. It was reported to be chondromyxoid fibroma.

**Figure 9:**
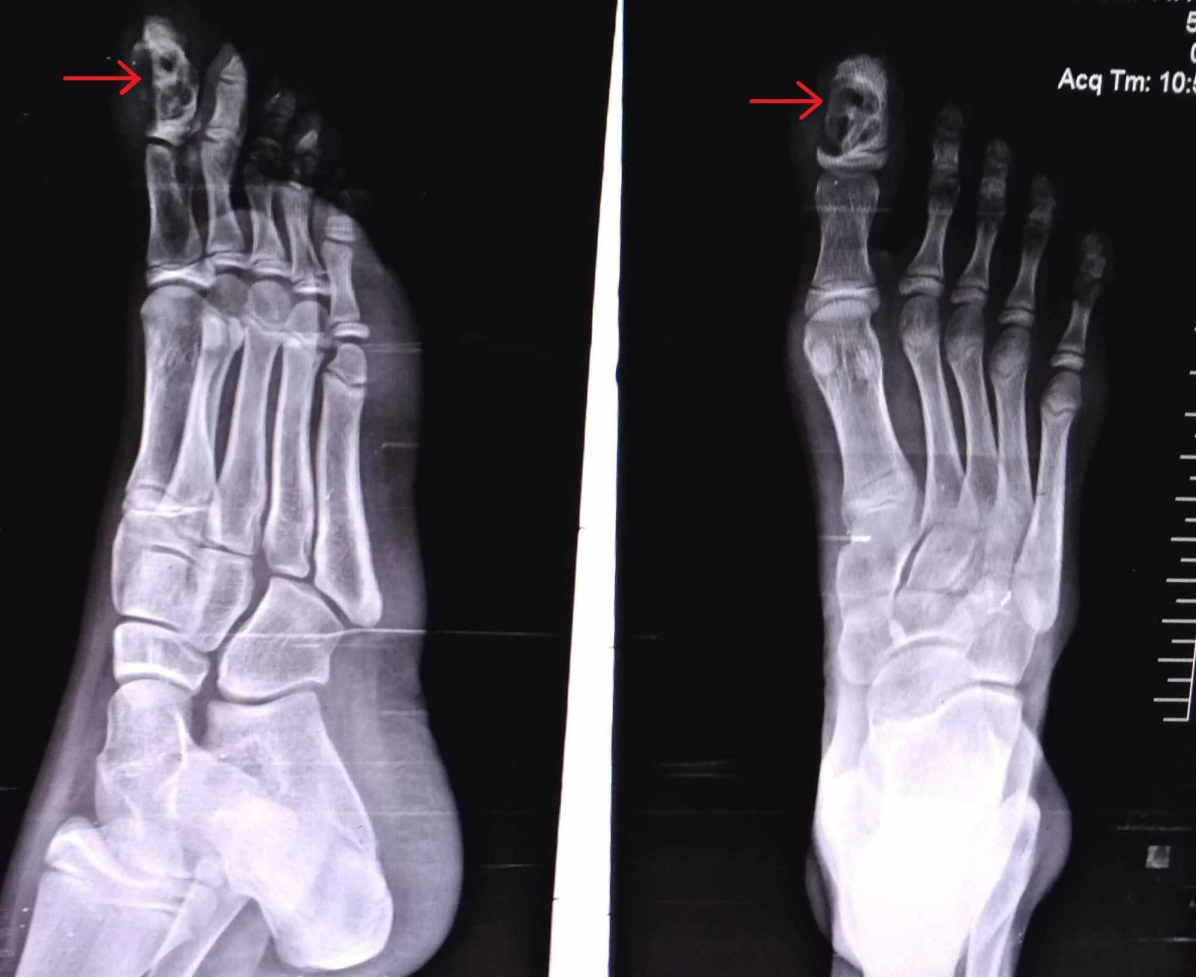
Anteroposterior and lateral view X-ray of the right foot The image shows recurrence of the tumor in the great toe

The patient presented six months later with complaints of pain in the great toe. X-ray right foot showed a lytic lesion in the distal phalanx of the great toe with well defined sclerotic and scalloped margins, coarse bony trabeculations, and without cortical expansion (Figure 9).

Since the tumor recurred, complete excision of the tumor with bone grafting was planned.

**Figure 10:**
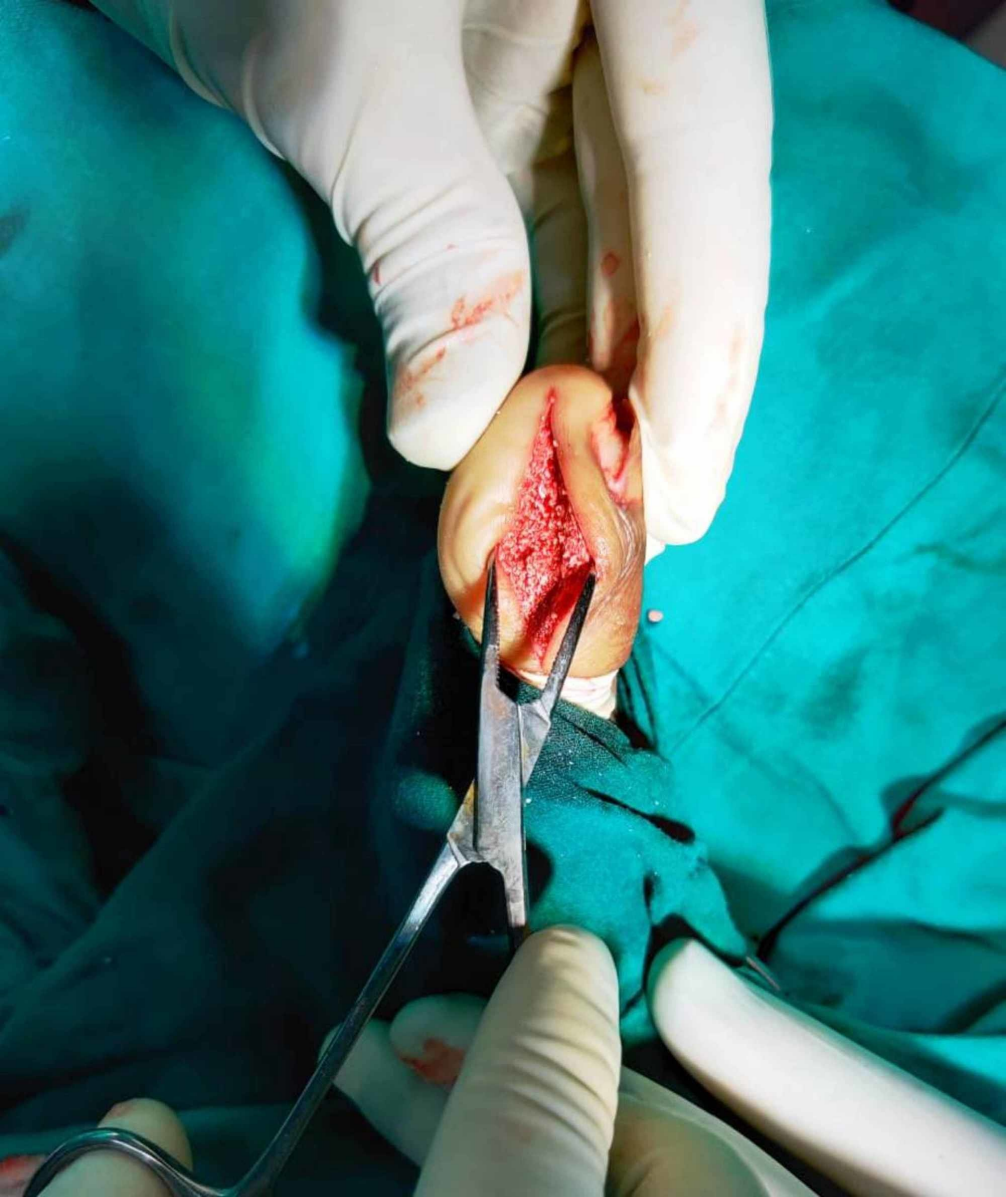
Defect filled with synthetic bone graft

**Figure 11:**
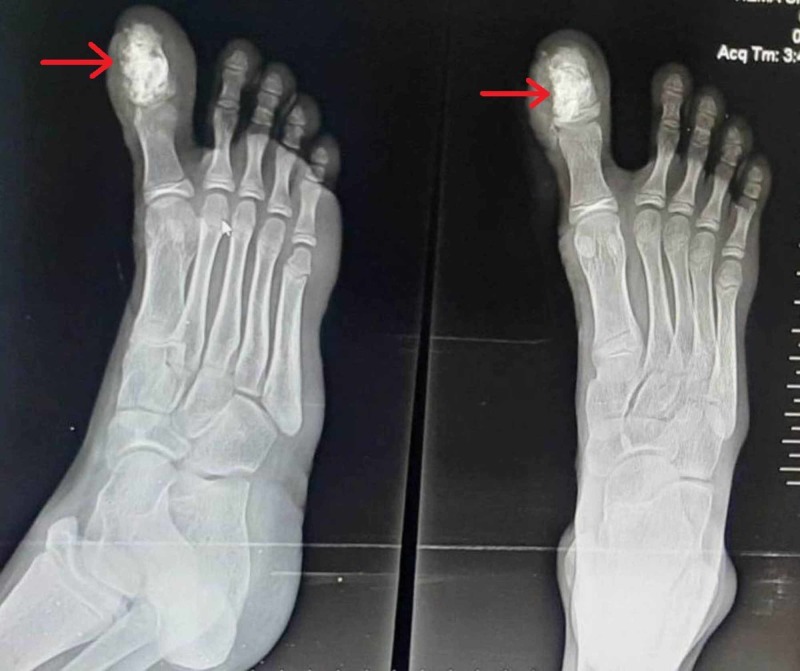
Post operative X-ray of the right foot The image shows defect filled with bone graft

The same medial approach was used again, and a cortical window was made to visualize the lesion. The cortex was found to be interrupted, and there was no involvement of the soft tissue. The lesion and the walls of the cavity were excised until healthy cortical bone was seen. The defect was filled using synthetic bone graft (G bone) as shown in Figures 10-11.

**Figure 12:**
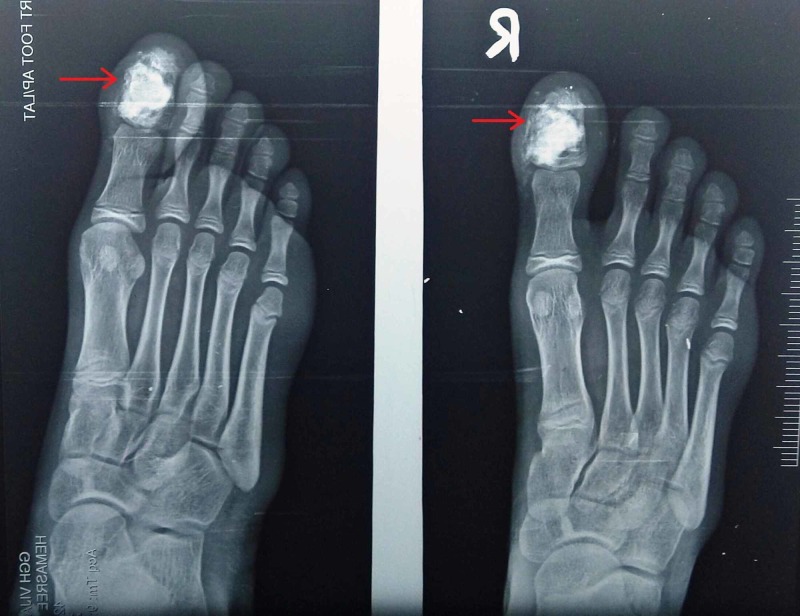
X-ray at six months follow-up The image shows incorporation of the graft

**Figure 13:**
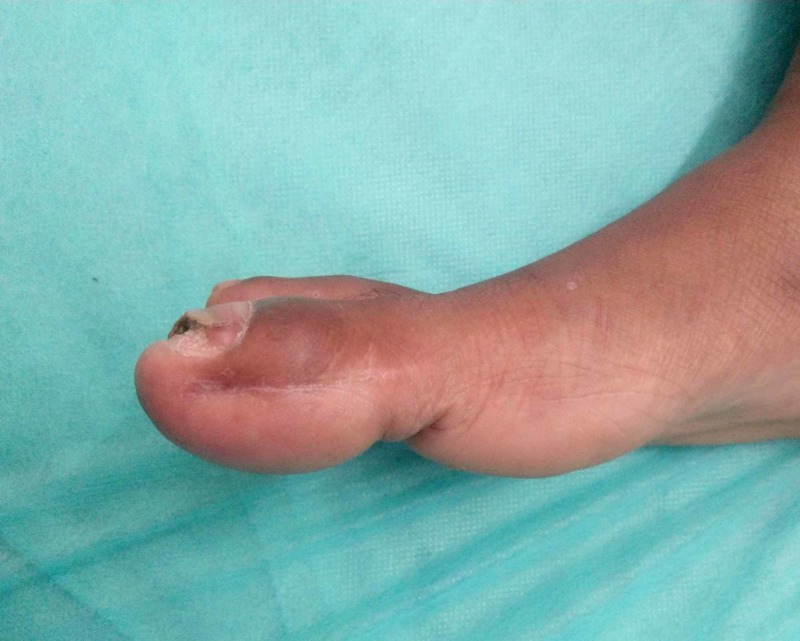
Surgical scar at six months

At six months follow up, the patient did not complain of pain, and X-rays showed signs of bone formation with incorporation of the graft (Figure 12). Scar had healed well (Figure 13).

## Discussion

Chondromyxoid fibroma is a rare benign tumor of cartilaginous origin. It was first described by Jaffe and Lichtenstein in 1948 [4]. It is frequently seen during the second or third decades of life. Metaphysis of long bone is the most common site [2]. The reported incidence of occurrence of the lesion in the short tubular bones of feet is 5% and is commonly seen in adults, i.e. during the third decade [5]. 

Radiologically, it is seen as an eccentric lesion in metadiaphysis of long bones and as a central lesion involving the entire width of the bone in short tubular bones. The lesion is usually surrounded by well-defined scalloped margins with intralesional trebaculations. Cortical thinning and expansion are seen. However, periosteal reaction is rare [6,7]. The lesion is usually confined to the metaphysis but can occasionally erode the growth plate to involve the epiphysis like in our case. This may also cause growth disturbances in the toe.

Definitive diagnosis can be made only on histopathology. The typical histopathology involves a lobular arrangement of stellate cells in a myxoid and chondroid background. The lobules are hypocellular with hypercellular fibrous septae. Osteoclast like cells can be seen in the peripheries [5].

Differential diagnoses

Chondrosarcoma: Chondromyxoid fibroma is a low-grade tumor, which may mimic chondrosarcoma. Radiologically extensive and irregular cortical destruction with soft tissue involvement is seen in chondrosarcoma. Histologically, both of them share the lobular pattern, myxoid stroma, and peripheral cellularity with nuclear atypia. However, chondrosacrcomas are more monotonous, have larger lobules, show abundant myxoid ground substance and less fibrous component. Mature hyaline cartilage is more likely to be seen on chondrosarcoma [8]. Chondromyxoid fibroma must be distinguished from chondrosarcoma to avoid misdiagnosis and aggressive treatment. Conversely, chondrosarcoma lesions diagnosed as chondromyxoid fibroma were under treated, which resulted in a worse prognosis later on [9].

Simple bone cyst: Commonly seen in children and adolescents, and cannot be differentiated radiologically. Grossly, the cyst is lined by a thin fibrous membrane and filled with fluid. HPE of bone cyst shows fibrous tissue containing giant cells and hemosiderin-laden macrophages, which is not seen in chondromyxoid fibroma [10].

Enchondroma: Commonly seen in fingers and toes. HPE shows paucicellular cartilage with mottled calcification. Myxomatous and chondroid stroma surrounded by fibrous septa, which is typical of chondromyxoid fibroma, is absent [11].

Aneurysmal bone cyst: Both of them share similar radiological features such as cortical expansion with bubbly appearance. Histology helps in differentiating them [11].

Low-grade infections can mimic chondromyxoid fibroma in radiology. However, periosteal reaction is commonly seen in infections, and cortical breach is seen in chondromyxoid fibroma. Both of them can be distinguished histologically [2].

The treatment options include curettage alone or with bone grafting or cement, en bloc excision or amputation [10].

Curettage alone, without packing the defect results in recurrence in about 20%-80% of the tumors [5]. This is probably due to incomplete removal of the tumor where the pseudopod projection extending from the main tumor into the spongiosa (bony crevices) may be left behind after a simple curettage. Curettage with bone grafting or cementation has a much lesser recurrence rate of about 7% [12]. In lesions where the cortex is found to be involved, a thorough curettage will leave a defect. It has to be filled with bone grafting to prevent fracture and to facilitate weight bearing. Recurrence rates are even higher in children less than 15 years of age, like in our case, possibly due to reduced resistance of thin growing bone to the tumor [2].

Amputation may be considered in malignant cases and multiple recurrences [7]. Although rare, long-term follow up of patients is necessary to watch for malignant transformation.

Distal phalanx of the great toe is an uncommon location for chondromyxoid fibroma and only a few case reports can be found. Bahk et al. reported a case of right great toe swelling which was diagnosed initially as osteosarcoma [13]. Interphalangeal disarticulation was done and the specimen was sent for histopathological examination. A final diagnosis of pseudoanaplastic chondromyxoid fibroma was done. Mallya et al. reported a case of left great toe chondromyxoid fibroma for which curettage and bone grafting was done [14]. However, the lesion recurred eight years later. Amputation of the great toe was done. Kim et al. reported a case of chondromyxoid fibroma of great toe with abnormal histological finding of hypercellular lobules separated by hypocellular septae [15]. It was treated with metatarsophalngeal disarticulation. Chang et al. reported a case of chondromyxoid fibroma of the great toe which was treated with amputation [16]. Majority of the reported cases treated the tumor by amputation of the toe. We treated the lesion with curettage and salvaged the toe.

## Conclusions

Chondromyxoid fibroma is an extremely rare neoplasm of the bone. There are no specific radiologic features, and histopathology provides a definitive diagnosis. It should be considered in the differential diagnosis of a solitary bone lytic lesion that has well-defined scalloped margins, and differentiated from other tumors, especially from chondrosarcoma to avoid aggressive treatment. To the best of our knowledge, our case of chondromyxoid fibroma of distal phalanx of the toe is the first in the literature to be reported that occurred before the closure of physis, as it is commonly seen among adults in short tubular bones.
